# Editorial

**Published:** 1872-03

**Authors:** 


### Editorial.

### *Order.*

To avoid delay in the present No., Original Communications have been placed after Selections. Correspondents are respectfully urged to hand in their favors on or before the 10th of the month.

### *Bogus Diplomas.*

It is evidence of a gratifying improvement in the public *morale*, that the legislature of Pennsylvania, on proof that certain so-called medical colleges had been engaged in the disreputable and infamous practice of selling diplomas, promptly repealed their charters.

### *Selling Practice.*

On another page will be found a legal decision which is of interest to physicians selling out their business, with a proviso that they will not continue in practice in the same locality. Such contracts are decided void, as being contrary to public policy—the community being entitled to every man's industry and skill.

In the case decided, the testimony shows that the venereal specialty ranks next, in its profitable results, to the womb business. As we are thoroughly "regular" ourselves, we will not undertake to say which branch of the two is the most respectable.

*Important to Sellers of Practice.*

An entirely new question in its scope in this State was on Saturday decided by Chief Justice Williams, of the Circuit Court. The question is, When does a contract in restraint of trade become void? The question arose on the chancery proceeding of Charles C. Jerome against Charles Bigelow. The opinion was as follows :

Complainant and defendant both profess to be physicians in the city of Chicago. Prior to May, 1870, the defendant had been practicing as a physician, and more particularly as a "specialist," and had secured in this and other States a considerable professional business. On the 10th of May, 1870, complainant and defendant entered into an agreement, by which the defendant stipulated, upon the consideration of $40,000, to be paid by complainant, in semi-annual payments of $2,000 each for ten years, to license and permit the complainant to enter in and take possession of his office in Chicago, and the goods and chattels therein, and to carry on the business in the special department in which defendant was then engaged, in the name and under the patronage of the defendant, from the 5th day of July, 1870, for the period of ten years thereafter, and that until the expiration of said ten years defendant would not, in his own name or otherwise, prosecute his profession in its general branches, nor in the specialty in which he had been engaged in the city of Chicago, nor within a radius of 500 miles thereof.

By a supplemental agreement, Bigelow is to take one-half of the net profits of the business instead of the payments agreed to be made by the original contract.

The defendant, after the fire, having resumed the practice of said specialty in the city of Chicago, the complainant, on the 18th of October last, filed his bill in this court, setting up the contract above referred to, alleging that he had performed his part of it, and had paid defendant under it several thousands of dollars, and praying that defendant might be restrained from engaging in and practicing the profession of medicine in the State of Illinois, or anywhere in the Northwest, for the remainder of the unexpired term of ten years.

The most important question raised upon the argument of this case is as to the validity of the contract so entered into on the 10th day of May, 1870, the defendant insisting that the contract is void, for the reason that it is in restraint of trade, and therefore against public policy.

The decisions upon this question run through a period of more than 450 years. The first reported case was A. D. 1415, in the reign of Henry V., when English jurisprudence was in its infancy, and when judicial manners were so rude that an English Judge accompanied the delivery of his opinionwith threats and blasphemy. The rule of law, as then stated, and long thereafter maintained, has been modified by the advancing civilization of the ages and the manners of the forum still more.

The law as it now exists may be thus stated : That the legal presumption is against every contract in restraint of trade, whether that restraint be general or partial ; that every contract for a general restraint of trade is absolutely void ; that while the presumption is against a contract in partial restraint of trade, such presumption is capable of being rebutted, and such contracts are often held valid, provided they are reasonable, and upon adequate consideration.

[The Court here cited the more important American decisions sustaining his statement of the law.]

Thus far there is no difficulty in determining what is the law, for not only is it so declared by the American Courts, but the uniform current of English decisions for more than 150 years, since the leading case of Mitchell *v.* Reynolds, (1 Peere Coms. 181), is in the same direction. But what shall be regarded as a general and what a partial restraint of trade, and what partial restraints shall be held valid and what void, are questions not so easily determined.

The vital question in reference to partial restraints of trade is, whether they are reasonable or unreasonable. If, in the opinion of the Court, the restraint has been greater than the protection of the party who seeks the benefit of the restriction requires, such restriction has been held unreasonable, and the contract void. Under the law as thus declared, each case of partial restraint must be decided with reference to its own particular circumstances, and the reasonableness or unreasonableness of the restriction being a question for the Court, it is not strange that different decisions have been made in cases where the facts were substantially the same. The principle controlling the case is well settled; the only difficulty is in its application.

Almost all the cases which I have had the opportunity of examining, whether English or American, are those in which the restriction has been confined to less territory than the kingdom or the State. Is a restriction co-extensive with the kingdom, in Great Britain, one which, by their Courts, would be regarded as general or total? In the case of Homer *v.* Ashford (3 Bingham, 326), Chief Justice Best, in delivering the opinion of the Court, said: "Any deed by which a person binds himself not to employ his talents, his industry, or his capital, in any useful undertaking in the Kingdom, would be void." Similar expressions are used in other English cases, but in none of them, except in the case in 3 Beavan, was the precise point before the Court; for, in none of them to which my attention has been called, with the above exception, was the restriction co-extensive with the territory of England.

In the case last referred to, Lord Langdale, Master of the Rolls, held that an agreement on the part of certain attorneys not to practice in Great Britain for the period of twenty years, without the consent of the attorney to whom they had sold their business, was not an unreasonable restriction, and that the contract was valid. This case, perhaps, goes further than any English case, and the correctness of it has been questioned by a learned English annotator. The English decisions appear to have assumed that a restraint, co-extensive with the kingdom of Great Britain, would have been void, and many of the expressions of the judges in their opinions, are to this effect, but generally they are mere dicta.

What shall be a general or total restriction of trade in America? Whether it shall be a restriction co-extensive with the particular State in which the contract is made, or with the boundaries of the United States, has not often been raised in our courts.

[The Court cited several authorities, to the effect that a total restraint of trade, as that a man will not pursue his occupation or carry on business anywhere in a State, is void.]

I have found no American case which holds that a contract in restraint of trade, which is co-extensive with State jurisdiction, is valid, while there are two decisions and many dicta which sustain the opposite view. The only English case which holds that a restriction extending throughout Great Britain is valid, is the one in 3 Beavan, referred to.

It is because the law protects all branches of trade and business from monopoly that it has held general contracts in restraint of trade void, and each State is better fitted to protect the rights of its citizens in this regard than the General Government. It is a matter pertaining to the municipal laws of each State, and each State in the Union is independent of every other as to these laws. It is for the interest of each State to protect and encourage trade, commerce and business within its own particular jurisdiction. Reason, as well as the weight of authority, is in favor of the view, that for the purpose of determining whether or not a restraint is general, reference is to be had to the boundaries of the State, and not of the United States.

The restriction in the case at bar prevented the defendant from practicing his profession generally or in the specialty in which he had been engaged in Chicago, or within a radius of 500 miles from this city, which by a further provision of the contract, was to be construed "to include all the Western and Northwestern States and Territories."

The defendant was thus excluded from practice, not only in the State of his residence, but also throughout adjoining States and Territories, containing an aggregate population of several millions, and he was not only restrained from practicing the particular specialty to which the contract immediately refers, but every branch of his profession. Under no decision, English or American, which I have examined, has such a contract been held valid. It is unreasonable, for it was far more of a restriction than was necessary to protect the interests of the complainant.

It is true, that it appears by the pleadings and evidence that under the name of "Charles Bigelow" the defendant was doing a large business as a specialist in Chicago, and also by a correspondence conducted with parties scattered all over the United States, yet it can hardly be contended that, in order to protect the interests of the complainant as a specialist in Chicago, it was necessary that the defendant should refrain from entering into a general practice as a physician in Minnesota, Kansas, and Colorado, and the yet more remote portions of the West and Northwest.

The contract is, therefore, void, because it is an unreasonable restraint. Inasmuch as the contract is void at law, the complainant is not entitled to any relief in equity. The bill will be dismissed.

---

CHICAGO, ILL., April 5, 1871.

DEAR PROFESSOR—

If a man commits suicide now, he is said to be afflicted with temporary insanity; if he shoots some one else, the law declares him to have been temporarily insane. I would invoke the common sense of the profession through your columns, on this matter.

Nearly all men know occasions during their lives when death would not seem unkind. Some men have never any great love for life; if then the temptation presents itself, reinforced perhaps by a sudden loss or so, to end the grand struggle, the proposition may be submitted to debate, and if the party most concerned can divest himself of that "fear of something after death," what is there remaining to hinder him though he be a rational party, from resorting to the hackneyed or seeking the unique in the way of quieting tissue metamorphosis forever?

If sleeplessness and perturbation result from too close a contemplation of the intended proceedings, the party is said to exhibit all the signs of insanity. Now this condition may be *called* insanity, but the party should be considered responsible for the steps by which he has *reasoned* himself into it.

Very respectfully, INKE MARKS.

